# LONG TERM OUTCOMES OF BIOMATERIAL-MEDIATED REPAIR OF FOCAL CARTILAGE DEFECTS IN A LARGE ANIMAL MODEL

**DOI:** 10.22203/eCM.v041a04

**Published:** 2021-01-07

**Authors:** M.L. Sennett, J.M. Friedman, B.S. Ashley, B.D. Stoeckl, J.M. Patel, M. Alini, M. Cucchiarini, D. Eglin, H. Madry, A. Mata, C. Semino, M.J. Stoddart, B. Johnstone, F.T. Moutos, B.T. Estes, F. Guilak, R.L. Mauck, G.R. Dodge

**Affiliations:** 1 Acute Cartilage Injury Consortium, AO Exploratory Research Collaborative Research Program, Davos Platz, Switzerland; 2 Translational Musculoskeletal Research Center, CMC VA Medical Center, Philadelphia, PA, USA; 3 Department of Orthopaedic Surgery, University of Pennsylvania, Philadelphia, PA, USA; 4 AO Research Institute Davos, Davos Platz, Switzerland; 5 Centre of Experimental Orthopaedics, Saarland University Medical Centre, Homburg/Saar, Germany; 6 School of Pharmacy, University of Nottingham, UK; 7 Department of Chemical and Environmental Engineering, University of Nottingham, UK; 8 Tissue Engineering Laboratory, Bioengineering Department, IQS School of Engineering, Universitat Ramon Llull, Barcelona, Spain; 9 Department of Orthopaedics and Rehabilitation, Oregon Health & Science University, Portland, OR, USA; 10 Cytex Therapeutics, Durham, NC, USA; 11 Washington University and Shriners Hospitals for Children, St. Louis, MO, USA

**Keywords:** Cartilage defects, large animal models, biomaterials, scaffolds, long-term outcomes, cartilage biomechanics, tissue engineering, orthopaedics, articular cartilage repair

## Abstract

The repair of focal cartilage defects remains one of the foremost issues in the field of orthopaedics. Chondral defects may arise from a variety of joint pathologies and left untreated, will likely progress to osteoarthritis. Current repair techniques, such as microfracture, result in short-term clinical improvements but have poor long-term outcomes. Emerging scaffold-based repair strategies have reported superior outcomes compared to microfracture and motivate the development of new biomaterials for this purpose. In this study, unique composite implants consisting of a base porous reinforcing component (woven poly(ε-caprolactone)) infiltrated with 1 of 2 hydrogels (self-assembling peptide or thermo-gelling hyaluronan) or bone marrow aspirate were evaluated. The objective was to evaluate cartilage repair with composite scaffold treatment compared to the current standard of care (microfracture) in a translationally relevant large animal model, the Yucatan minipig. While many cartilage-repair studies have shown some success *in vivo*, most are short term and not clinically relevant. Informed by promising 6-week findings, a 12-month study was carried out and those results are presented here. To aid in comparisons across platforms, several structural and functionally relevant outcome measures were performed. Despite positive early findings, the long-term results indicated less than optimal structural and mechanical results with respect to cartilage repair, with all treatment groups performing worse than the standard of care. This study is important in that it brings much needed attention to the importance of performing translationally relevant long-term studies in an appropriate animal model when developing new clinical cartilage repair approaches.

## Introduction

Focal cartilage defects may arise as a result of mechanical trauma and disrupt the articular surface (Buckwalter, 2002). Focal cartilage defects can be painful, interrupting daily activities and decreasing quality of life (Heir et al., 2010). Given the poor intrinsic regenerative capacity of the tissue, left untreated, large full-thickness cartilage defects may increase in size, ultimately resulting in widespread degeneration of the joint surface and OA (Heijink et al., 2012). Currently, OA affects ≈ 14 % of the US adult population. Projections based on the increasing prevalence of OA predict that 25 % of the US adult population will be affected by the year 2030, underscoring the need for the field to develop more effective defect repair strategies that limit lesion progression and culminate in restoration of tissue and joint function (Web ref. 1).

To address this unmet clinical need, a number of focal defect repair methods have been developed, with MFX being the earliest and most commonly used clinically (Benthien and Behrens, 2010; Brittberg et al., 1994; Kon et al., 2013; Steadman et al., 2001). MFX involves perforating the subchondral plate to allow the flow of blood and MSCs from the subchondral marrow into the defect with the goal of inducing chondrogenesis. MFX can be performed in a single arthroscopic procedure, is less technically demanding than other cartilage repair procedures, and has favourable short-term outcomes (2-4 years follow-up) (Mithoefer et al., 2005). While these aspects have resulted in MFX becoming the first-line treatment for focal cartilage defect repair, the technique is not without drawbacks. In a long-term study (10-14 years follow-up) of MFX treatment by Solheim *et al.*, 39 % of patients required additional surgery and 46 % had poor clinical outcomes (Solheim et al., 2016). MFX and other marrow stimulation techniques result primarily in the formation of fibrocartilage, which differs from native hyaline cartilage in both biochemical composition and mechanical properties (DiBartola et al., 2016; Ebenstein et al., 2004). It is possible that the inferior mechanical properties of MFX-induced tissue result in degradation and failure over time, contributing to the high rate of revision surgeries and poor long-term outcomes.

The development of new techniques that better reproduce the properties of native hyaline cartilage are critical for lasting repair. Current innovative repair procedures rely on matrix- and cell-based approaches, including MACI™ or AMIC™. In MACI, autologous chondrocytes are harvested and cultured within a scaffold, prior to implantation into a cartilage defect. AMIC combines MFX with a scaffold in order to enhance chondrogenesis of MSCs entering the defect site. Both MACI and AMIC have shown superior clinical outcomes when compared with MFX alone (Brittberg et al., 2018; Volz et al., 2017).

The success of MACI and AMIC motivates the development of new biomaterials that can further enhance cartilage repair. One promising class of biomaterials is hydrogels, which can be tuned to mimic the native extracellular environment of cartilage and can enhance the chondrogenic capacity of MSCs (Spiller et al., 2011). Two such hydrogels that have shown promise *in vitro* and in preliminary small animal trials are HA and peptide-based materials (Bussmann et al., 2016; Johnstone et al., 2012; Mortisen et al., 2010; Seelbach et al., 2014). These hydrogels can both support matrix production and tissue maturation by chondrocytes and MSCs (Bian et al., 2013; Bosnakovski et al., 2006; Fernández-Muiños et al., 2015; Sechriest et al., 2000; Williams et al., 2003). However, the use of hydrogels *in vivo* can be challenging due to the low stiffness of these materials at the time of implantation. One strategy to address this issue is the development of composite scaffolds with a porous reinforcing component to provide structural support, and a hydrogel component to enhance chondrogenesis (Mohabatpour et al., 2016; Moutos and Guilak, 2008; Wang et al., 2016; Zhao et al., 2009). For example, woven 3-dimensional PCL scaffolds match the mechanical properties of native cartilage and can be infiltrated with chondroinductive hydrogels (Moutos and Guilak, 2010).

While many such materials and combinations have been evaluated *in vitro* for matrix formation, ultimately the *in vivo* evaluation of new cartilage repair scaffolds is a critical step in their translation into clinical practice. A variety of animal models of cartilage injury exist and have been utilised to study cartilage repair, including small animals and large animals (Cook et al., 2014). Large animal models can provide particularly valuable insight, as their joints better approximate the anatomical, biomechanical, and biochemical environment of human synovial joints (Moran et al., 2016). While *in vivo* studies are a critical step in translation, a recent systematic review showed varied results and that most studies were short term (< 3 months) with few common outcome measures evaluated (Pfeifer et al., 2015). While short term studies are more cost effective, long-term studies are essential to establish durability of technologies and are more relevant to the clinical scenario. Thus, the goal of this study was to evaluate 2 novel composite scaffolds combined with MFX in a clinically relevant large animal model. Working within an international consortium, a stepwise approach was taken from initial studies showing the ability of these scaffold designs to support chondrogenesis *in vitro* to a 12-month structure-function study using the established minipig cartilage defect model (Bonadio et al., 2017; Friedman et al., 2018; Recha-Sancho et al., 2016; Venkatesan et al., 2018). Here, the results are reported that were counter to the initial short-term findings (Friedman et al., 2018), which supports the importance of performing these types of studies through longer, more clinically relevant time points.

## Materials and Methods

### Animal study

**Fig. 1.**
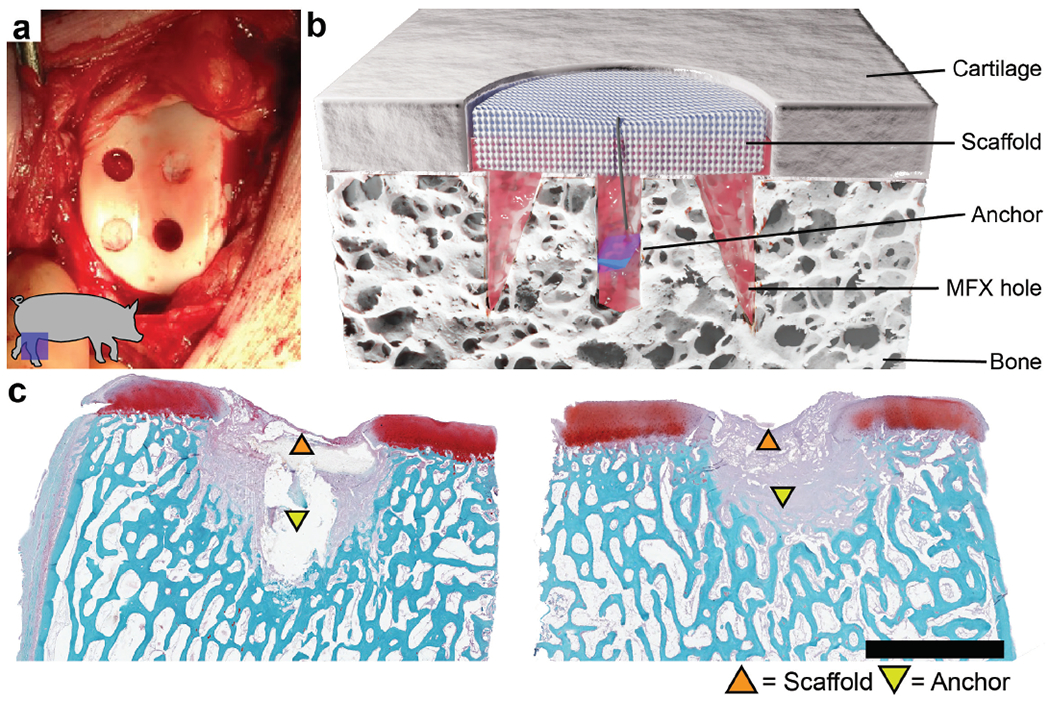
Defect creation, scaffold implantation, and early term outcomes. (**a**) Four full-thickness chondral defects (4 mm in diameter) created in the femoral trochlea of the stifle joint of a Yucatan minipig. (**b**) Cross-section schematic demonstrating scaffold placement within the defect with subchondral bone anchor. (**c**) Histological images from a pilot study (Friedman et al., 2018) demonstrating scaffold retention with a subchondral bone anchor at a 6-week timepoint in 2 separate defect specimens. Scale bar = 4 mm.

All animal procedures were approved by the Institutional Animal Care and Use Committee (IACUC) at the University of Pennsylvania. 12 skeletally mature (age 12-16 months at beginning of study) Yucatan mini pigs (Sinclair Bioresources, Auxvasse, MO, USA) underwent unilateral stifle joint surgery using a minimally invasive open approach (Bonadio et al., 2017). 4 full-thickness chondral defects were created in the trochlear groove using a 4 mm biopsy punch. To mimic clinical defect preparation, a curette was used to remove the cartilage within the biopsy margins down to the level of the subchondral plate. Woven PCL scaffolds were fixed within the defect using a subchondral anchor (Mitek Microfix, Depuy, Raynham, MA, USA) (Friedman et al., 2018; Moutos and Guilak, 2010) (Fig. 1). Scaffolds were placed following MFX only, MFX with injection of a self-assembling peptide hydrogel (RADA, PuraMatrix, Corning Life Sciences, Corning, NY, USA), MFX with injection of a thermogelling HA hydrogel (AO Research Institute, Davos, Switzerland), or injection of BMA, from the proximal tibia, without MFX (Bussmann et al., 2016; D’Este et al., 2012; D’Este et al., 2016; Madry et al., 2017; Mortisen et al., 2010). RADA, HA, and BMA were injected into the empty defects, followed by placement of PCL scaffolds and a second injection into the scaffold itself. MFX-only and anchor-only groups were included as controls (Table 1). In defects that received MFX, 3 equidistant holes were placed near the defect margin, using an awl (approximately 0.7 mm diameter by 2 mm deep). These 6 treatment groups were randomised across 48 defects (*n* = 8/treatment group). All surgical procedures were performed under anaesthesia with sterile technique. Animals were allowed full weight-bearing immediately following recovery and were housed independently. Animals were euthanised ~ 1 year after the implantation surgery. Following dissection of the operative stifle joint, a photograph was taken, and a saw was used to produce 1 cm^3^ osteochondral units containing the chondral defect. Control samples were collected from the trochlea of the non-operative limb.

### Mechanical testing

All samples were stored in PBS with protease inhibitor cocktail (Roche Complete, MilliporeSigma, Burlington, MA, USA) at 4 °C and underwent indentation testing within 24 h of collection. Immediately before indentation testing, samples were potted in polymethyl methacrylate (Ortho-Jet, Lang Dental, Wheeling, IL, USA) such that the bone was submerged and held in place. After sample potting, cartilage remained submerged in PBS/protease inhibitor for the duration of the indentation test. All indentation testing was performed on an Instron (Instron, Norwood, MA, USA) equipped with a 10 N load cell and a 2 mm diameter spherical indentation tip (Meloni et al., 2017). Testing consisted of 4 steps of 10 % strain (applied at 0.1 %/s), followed by a 600 s hold to allow the sample to reach equilibrium. A custom MATLAB® program (MathWorks, Natick, MA, USA) was used to calculate equilibrium modulus. Briefly, data from the third step of the indentation test was isolated, and the equilibrium load was calculated as the load at the end of the 600 s relaxation, minus the initial load. Equilibrium deformation was similarly calculated. The equilibrium deformation was divided by the initial cartilage thickness to determine the equilibrium strain, and the equilibrium force was normalised, to an estimate of the final contact area of the indenter with the sample, to determine the equilibrium stress. Modulus was defined as the stress divided by the strain.

### μCT and histology

Following mechanical testing, samples were fixed in 10 % neutral buffered formalin and underwent μCT scanning (Scanco μCT50, Scanco Medical, Southeastern, PA, USA). Scans were conducted utilising the following parameters: 3000 projections, 900 ms × 5 exposures/projection, voltage: 45 kVp, current: 133 μA, isotropic voxel size: 6 μm. Cylindrical volumes of interest surrounding the defect were defined, starting at the subchondral plate (6 mm diameter × 5 mm deep). The Scanco evaluation software was used to analyse standard bone morphometrical parameters, including BV/TV, connectivity density, trabecular number, trabecular thickness, trabecular spacing, and mean BMD.

Following μCT imaging, samples were decalcified (Formical 2000, StatLab Medical Products, Columbia, MD, USA) for approximately 1 month and processed for paraffin-wax histology. Sections from the midplane of each defect (7 μm thick) were cut and stained with haematoxylin and eosin and safranin O/fast green (safO/FG).

### Scoring and data analysis

**Fig. 2.**
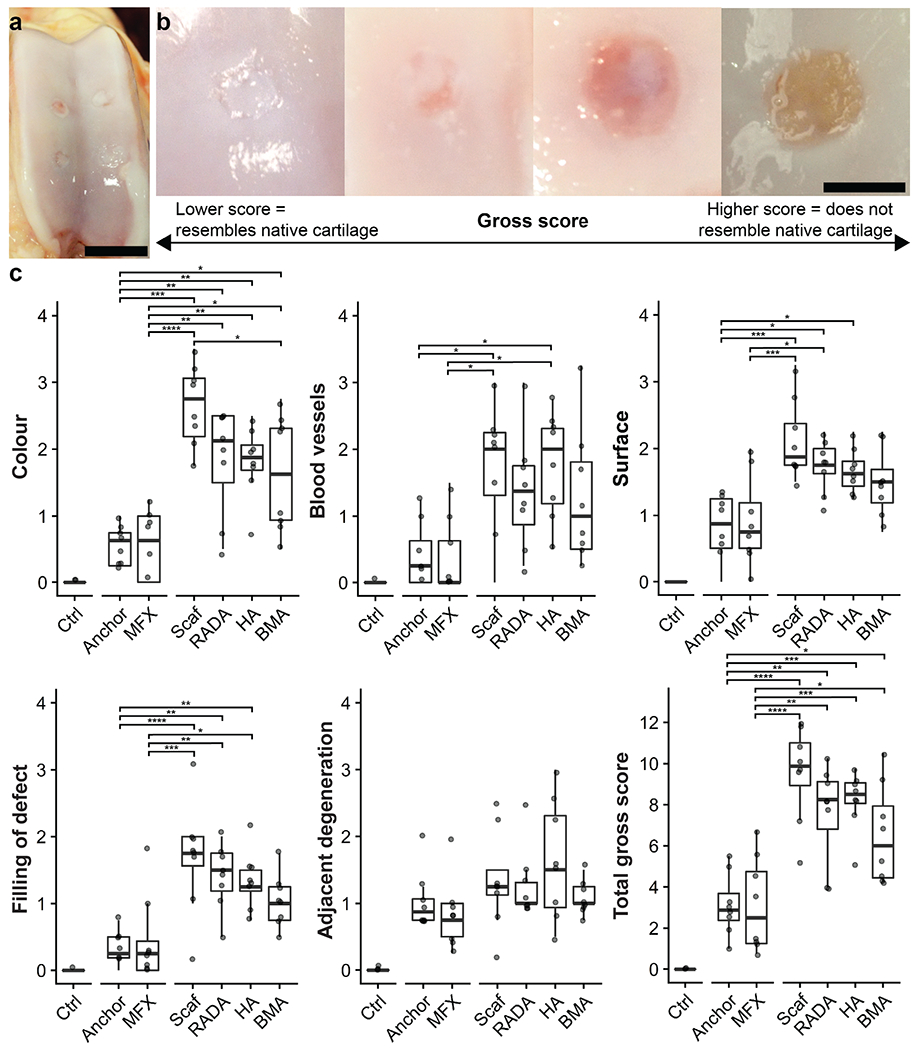
Gross images and semi-quantitative scoring of cartilage repair. (**a**) Representative image of an operative stifle joint taken at the time of euthanasia. Scale bar = 20 mm. (**b**) Grossly, defects demonstrate a range of repair quality. Scale bar = 4 mm. (**c**) Gross scoring results from the Goebel scoring method (Goebel et al., 2012). Individual parameters are scored 0-4 for a maximum total score of 20. Lower scores indicate higher quality repair tissue. *p < .05, **p < .01, ***p < .001, ****p < .0001. Plots show median and interquartile range.

**Fig. 3.**
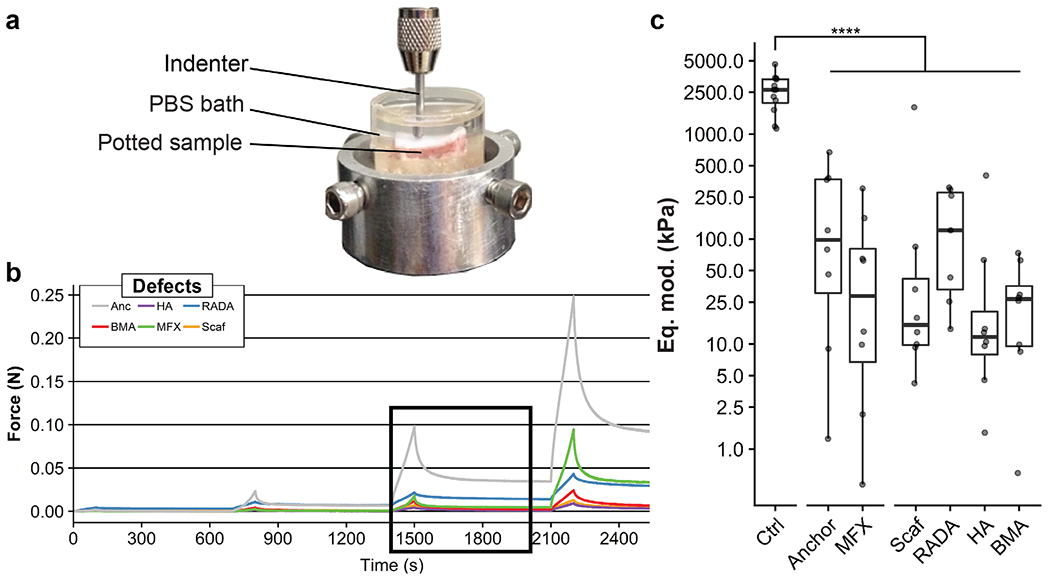
Mechanical properties of regenerative tissue. (**a**) Testing platform for cartilage indentation testing. (**b**) Representative force *vs.* time curves for defect samples. Box indicates third step of stress relaxation test. (**c**) Estimated equilibrium moduli (Eq. mod.) (displayed on a log_10_ scale) were calculated from the 3^rd^ step of each curve. All treatment groups had a significantly (*p* < 0.0001) lower equilibrium modulus compared with healthy cartilage control. Plot shows median and interquartile range.

**Fig. 4.**
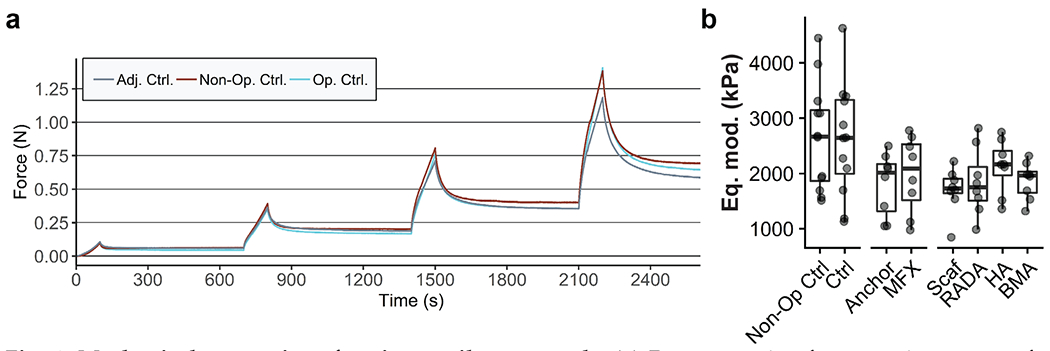
Mechanical properties of native cartilage controls. (**a**) Representative force *vs.* time curves for indentation tests of cartilage adjacent to experimental defects (within 2 mm), proximal to the defects within the operative joint, and from the femoral trochlea of the non-operative limb. (**b**) No differences in equilibrium moduli (Eq. mod.) were observed between the various controls. Plot shows median and interquartile range.

**Fig. 5.**
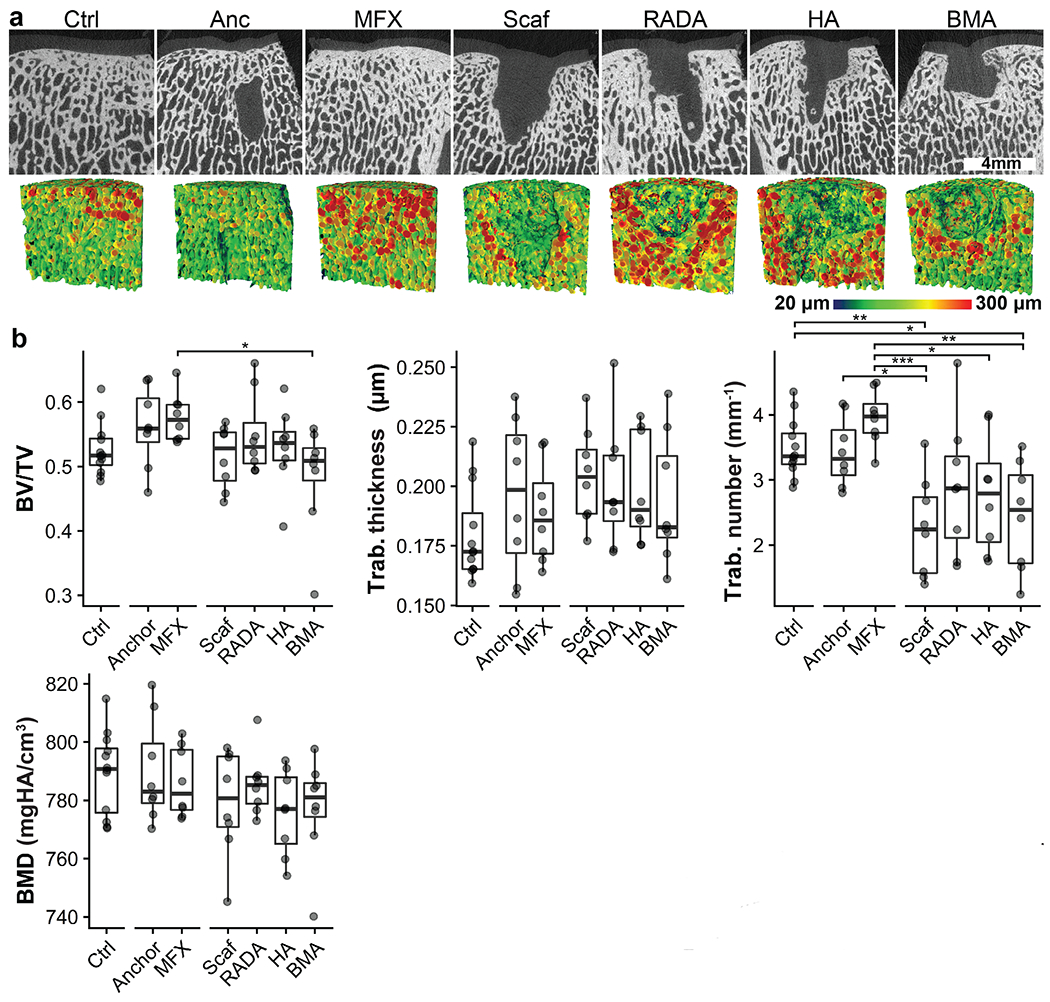
μCT analysis. (**a**) Top row: mid-plane slice from a representative sample in each group. Bottom row: mid-plane of renderings of a 6 mm diameter × 5 mm deep volume of interest used to analyse bone morphometry. Heatmap shows trabecular (Trab.) thickness. (**b**) Results of quantitative bone morphometrical analysis. *p < .05, **p < .01, ***p < .001. Plots show median and interquartile range.

Gross images taken at the time of euthanasia were assessed by 4 blinded scorers using a macroscopic scoring system for articular cartilage repair (Goebel et al., 2012). In addition, SafO/FG stained sections were assessed using a modified ICRS II scoring system (Mainil-Varlet et al., 2010). Repair tissue was scored on the following parameters: matrix staining, surface architecture, cell morphology, superficial zone clustering, deep zone clustering, subchondral bone involvement, basal integration, lateral integration, surface assessment, deep assessment, adjacent assessment, and overall assessment. Each parameter was scored on a continuous scale from 0 (poor defect repair) to 100 (normal articular cartilage) by 5 blinded scorers with expertise in cartilage histomorphology. For each defect, scores were averaged and plotted by treatment group. All quantitative data were analysed with GraphPad Prism (version 8.4.3 for macOS, GraphPad Software, San Diego, CA, USA). Results from gross scoring, mechanical testing, μCT, and histological scoring were compared using one-way ANOVA with Tukey’s correction for multiple comparisons (*α* = 0.05). Plots in Figs. 2–5 were generated using the ggplot2 plugin for R (Web ref. 2).

## Results

### Surgical outcomes and gross scoring

All animal procedures were performed without complication, and animals were weight-bearing within 2 h following surgery. All 12 animals remained healthy for the duration of the study and were euthanised 1 year after surgical defect creation. At the time of euthanasia, all operative joints appeared healthy, with no oedema or erythema observed. Following dissection of the stifle joint capsule, defects were easily visualised in the femoral trochlea, and grossly displayed a wide range of repair quality (Fig. 2). In general, defects without a woven PCL scaffold implanted appeared to have a higher volume of opaque tissue infill. Blinded semi-quantitative gross scoring supported this observation, with anchor-only and MFX groups scoring significantly better on most parameters. Adjacent cartilage appeared healthy in all groups and no significant differences were observed in this parameter.

### Mechanical testing

Indentation testing was performed to assess the mechanical properties of the repair tissue as well as the native cartilage adjacent to the defects. All defect groups had significantly lower moduli than healthy native cartilage. No significant differences were observed between any of the defect groups (Fig. 3). Indentation testing of cartilage adjacent to the defects showed no difference in mechanical properties when compared with native healthy cartilage from the non-operative limb (Fig. 4).

### μCT

μCT analysis was performed in order to assess the structural properties of the subchondral bone. Anchor-only and MFX-treated defects appeared to have an intact subchondral plate, with increased trabecular thickness near the osteochondral interface. A radiolucent region was observed in anchor-only samples and was assumed to be the remnants of the resorbable anchor. In all scaffold containing samples, a large radiolucent volume was observed in the region immediately beneath the site of the chondral defect, demonstrating dramatic bone resorption. This resorbed volume was surrounded by a rim of thickened bone. Supporting these qualitative observations, quantitative analysis showed a trend toward decreased BV/TV and increased trabecular thickness, as well as a significant decrease in trabecular number amongst scaffold-treated groups (Fig. 5).

### Histology

**Fig. 6.**
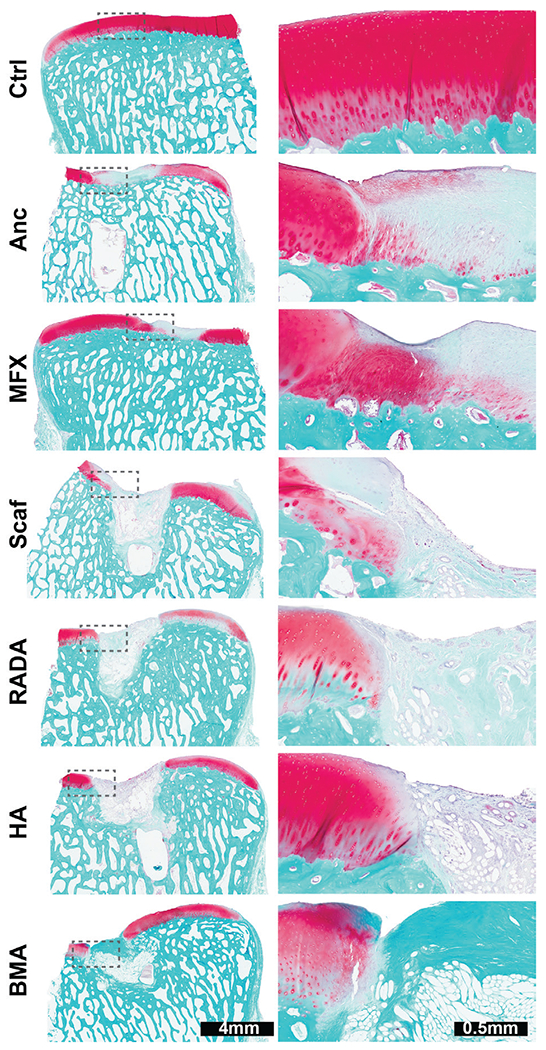
Safranin O/fast green staining. Safranin O/fast green staining of representative defects from each treatment group demonstrates marked subchondral bone resorption and limited safranin O staining of repair tissue.

**Fig. 7.**
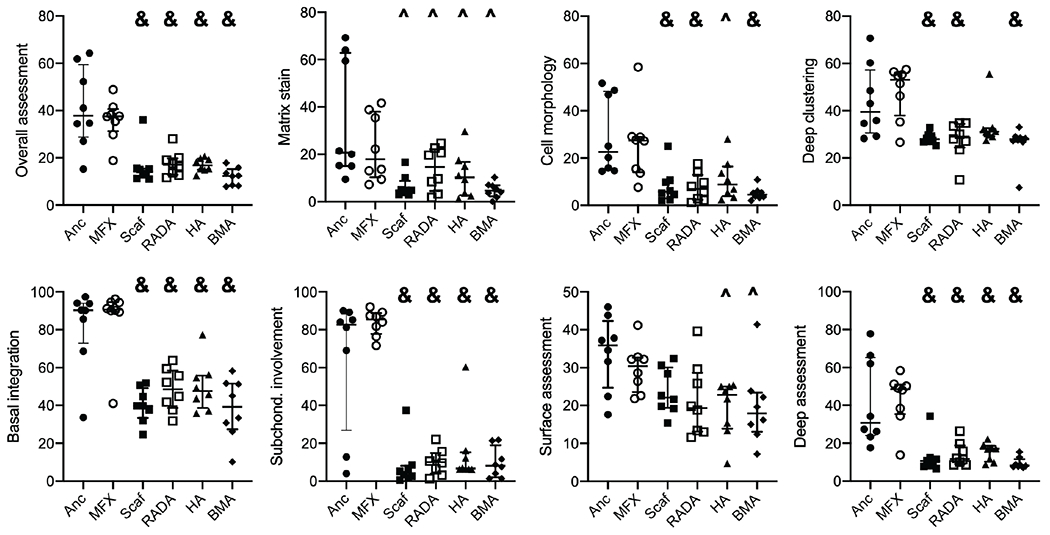
Blinded histological scoring. Significant differences were found in the overall assessment, matrix stain, cell morphology, deep clustering, basal integration, subchondral involvement, surface assessment, and deep assessment parameters. ^ = indicates significant difference from anchor group, & = significant difference from both anchor and MFX groups. Bars indicate median and interquartile range.

Histological analysis revealed substantial disruption to the subchondral plate and bone in all scaffold treatment groups. In most of scaffold-treated defects, the scaffold had subsided below the level of the adjacent subchondral plate. Anchor and MFX treated defects showed repair of the subchondral plate and less disruption to the subchondral bone. The plastic anchor was still visible at the time of sectioning but fell out of most blocks upon contact with the blade, generating a sectioning artifact in the tissue. In all groups, the repair tissue within the defect was fibrocartilaginous and exhibited limited safranin O staining (Fig. 6). Blinded scoring to assess the quality of repair tissue and surrounding tissue revealed no significant differences between the anchor and MFX groups across all parameters. Scaffold treated defects generally scored significantly lower than MFX and anchor only controls (Fig. 7).

## Discussion

This study used a multidisciplinary approach in terms of study design and outcome measures to address the challenge of focal cartilage defect repair. The study evaluated long-term outcomes of novel woven PCL/hydrogel composite scaffolds and microfracture in a clinically relevant large animal model of cartilage injury. Short-term pilot studies performed by the group showed reasonable cartilage repair outcomes (Friedman et al., 2018). Unfortunately, the promising short-term results did not persist at the 12-month time point, where no benefit was observed for woven PCL alone or either of the PCL/hydrogel composite scaffolds. In fact, scaffold placement resulted in reduced healing compared with the current standard of care treatment, MFX. Mechanical testing showed poorer properties for all treatment groups when compared with native cartilage controls. While the repair response of the MFX group was poorer than native, it was generally better than for defects treated with scaffold. This is likely due in part to the fact that scaffold treatment resulted in a substantial subchondral bone remodelling response, resulting in varying degrees of scaffold subsidence with overlying fibrocartilage formation. Having migrated to well below its initial implantation position at the articular surface, the scaffold was not able to function as designed to provide mechanical reinforcement to newly formed tissues.

The degree of subchondral bone remodelling observed in scaffold-treated defects was not observed in short-term pilot studies with animals of this age. Previous studies, utilising this animal model, found that the creation of a full-thickness chondral defect alone will evoke a remodelling response in skeletally immature animals, but that this bone remodelling decreases with skeletal maturity (Fisher et al., 2015; Pfeifer et al., 2017). These findings informed the decision to utilise skeletally mature animals in the current study. In control groups that did not receive a scaffold (MFX, anchor only), μCT and histological analysis did not show substantial subchondral resorption, and the subchondral plate remained intact. Furthermore, while the plastic anchor was present at the conclusion of the study, there was no evidence of the microfracture holes or the anchor drill hole in the MFX and anchor only groups, respectively, suggesting that the lytic response is associated with the presence of a scaffold. It is hypothesised that the untoward response to the woven scaffold may be a result of a lack of adequate fixation in the defect, as only the centre portion of the implant was tethered to the underlying bone. Since bone is a highly dynamic tissue that undergoes remodelling in response to hormonal, inflammatory, and mechanical stimuli (Sims and Gooi, 2008), attention was focused here for possible explanations of the lytic response. There are several possible aetiologies for the pathologic bone remodelling observed with scaffold treatment, including altered loading of the subchondral bone underlying the defect or diffusion of inflammatory factors from the synovial fluid into the bone (Zysk et al., 2004). It is also well-documented with total joint arthroplasty procedures, that perioperative implant loosening, micro motion, and microscopic abrasion are known to be some of the principal drivers of aseptic loosening, promoting both an inflammatory response and, in turn, an aggressive osteolytic response (Abu-Amer et al., 2007). It is possible that the lytic response observed in the current study occurred in the first several months following implantation and then stabilised in the defect, as is evident by the ECM tissue accumulation within the scaffold and the degree of integration with host tissues (Fig. 6). This may also be the reason why the presence of inflammatory cells in our microscopic histological analysis was not observed. Furthermore, recent studies using a similar scaffold that was rigidly press-fit in a different animal model (femoral head of the canine hip) showed excellent integration and no subsidence (Web ref. 3). In this regard, bolstering the rigidity and the fixation of the 3D woven scaffold are in order for future iterations of the implant. Furthermore, these findings indicate that the choice of an animal model, including factors such as species, age, and specific joint location, may influence the long-term success of tissue-engineered cartilage repair.

While the experimental treatment group did not perform as expected, this study still had several important outcomes. First, these results illustrate the importance of performing long term and large animal studies. While short term studies are important for determining degradation kinetics and biocompatibility of new biomaterials, there is no substitute for long term studies when assessing the quality and durability of repair tissue. This study also provides additional evidence of the poor quality of MFX repair tissue. Our finding that MFX repair tissue is predominantly fibrocartilaginous is consistent with both the clinical literature and other large animal studies (Breinan et al., 2000; DiBartola et al., 2016; Erggelet et al., 2009). Furthermore, the mechanical testing results showed that MFX repair tissue has a significantly lower modulus than native cartilage at a 1-year time point. This finding suggests that there may be a discordance between the mechanical properties of MFX tissue and positive short-term clinical outcomes. It is possible that the covering of fibrocartilage is enough to alleviate pain and improve patient reported outcomes at early time points. In order to reduce the poor long-term outcomes of MFX, it will be necessary to improve upon the weak repair tissue produced by this technique. In addition, this study underscores the need to perform multiple outcome measurements when assessing cartilage repair outcomes. For example, the MFX group scored significantly lower than scaffold groups in the gross scoring assessment (closer to native cartilage) but performed similarly to scaffold groups in mechanical testing. While this study included a number of outcome measurements, there remains an unmet need for standardisation of large animal cartilage repair studies to facilitate cross-study comparisons and expedite translation of novel cartilage repair strategies (Pfeifer et al., 2015).

While any large animal study is challenged by sample size limitations, variations in response across donors were noted, with some animals in each group performing well, while others had limited regenerative responses. Notably, variability can come from a number of sources including genetic and epigenetic factors. All procedures were performed by the same surgeon and surgical team, and as such, the variability observed likely arises from differences in animal subjects. Nonetheless, an improved understanding of the causes of variability in the repair response will provide new insights into the development of improved techniques for cartilage regeneration.

## Conclusions

This study built upon reasonable short-term findings to test an ambitious long-term study with multiple outcome measures. While numerous studies have explored cartilage repair in a variety of animal models, typical time frames are short and may not support the relevance for structural repair in the long term. This study also points out the need for standardised outcome measures that can be applied across the field of study that address both structure and function. In addition, this study emphasises the importance of team science in translating novel cartilage repair strategies. Regardless of outcome, the complexity and scale of a large animal preclinical study requires expertise that can only be achieved through collaboration.

## Figures and Tables

**Table 1.** Group characteristics.

Group (abbreviation)	*n*	Anchor	Microfracture
*Anchor only (Anchor)	8	Yes	No
*Miciofracture only (MFX)	8	No	Yes
PCL scaffold (Scaf)	8	Yes	Yes
PCL scaffold + RADA (RADA)	8	Yes	Yes
PCL scaffold + hyaluronan (HA)	8	Yes	Yes
PCL scaffold + bone marrow aspirate (BMA)	8	Yes	No

* = non-scaffold control group.

## List of Abbreviations

μCT: micro-computed tomography
AMIC: autologous matrix induced chondrogenesis
ANOVA: analysis of variance
BMA: bone marrow aspirate
BMD: bone mineral density
BV/TV: bone volume/total volume
ECM: extracellular matrix
HA: hyaluronan
MACI: matrix-associated chondrocyte implantation
MFX: microfracture
MSCs: mesenchymal stem/progenitor cells
OA: osteoarthritis
PBS: phosphate-buffered saline
PCL: poly(ε-caprolactone)
safO/FG: safranin O/fast green
